# The mechanisms crosstalk and therapeutic opportunities between ferroptosis and ovary diseases

**DOI:** 10.3389/fendo.2023.1194089

**Published:** 2023-07-27

**Authors:** Ying Yao, Bin Wang, Yanbiao Jiang, Hong Guo, Yulan Li

**Affiliations:** ^1^ The First School of Clinical Medicine, Lanzhou University, Lanzhou, China; ^2^ Department of Anesthesiology, The First Hospital of Lanzhou University, Lanzhou, China

**Keywords:** ovarian diseases, ferroptosis, iron metabolism, lipid peroxidation, therapeutics

## Abstract

Ferroptosis, a form of regulated cell death, was first defined in 2012. Ferroptosis mainly involves iron-driven lipid peroxidation damage of cells. This process is regulated by iron homeostasis, redox balance, lipid metabolism, glutathione metabolism, and various disease signaling pathways. Iron is one of the key mineral elements that regulate the physiological function of women and the development of ovarian tumors. Occurrence of Ferroptosis has some hidden dangers and advantages in ovary diseases. Some scholars have shown that ferroptosis of ovarian granulosa cells (GC) promotes the development of ovarian dysfunction and polycystic ovary syndrome (PCOS). Interestingly, drug-resistant ovarian cancer cells are very sensitive to ferroptosis, suggesting that pharmacological positive and negative regulation of ferroptosis has great potential in the treatment of benign ovarian diseases and ovarian cancer. This article aimed to assess how ferroptosis occurs and the factors controlling ferroptosis. Moreover, we summarize how ferroptosis can be used to predict, diagnose and target treatment ovary disease. Meanwhile, we also evaluated the different phenomena of Ferroptosis in ovarian diseases. It aims to provide new directions for the research and prevention of female reproductive diseases.

## 1 Introduction

Ferroptosis was originally defined as iron-mediated lipid peroxidation damage, which is closely related to the physiological and pathological mechanisms of the ovary. Although iron is a micromineral component, it is essential in various biological processes, including oxygen transport, energy metabolism, cell growth, and differentiation. Women have some unique physiological characteristics, such as excessive menstrual volume, increased maternal blood volume during pregnancy, fetal development, and postpartum blood loss. Therefore, adult women usually need more iron than men (1). However, iron is a ‘ double-edged sword ‘ and thus should be carefully used while taking measures to prevent iron deficiency. Iron overload can increase unstable iron content in the body. Excessive free active iron can cause lipid peroxidation of polyunsaturated fatty acids in the cell membrane in a non-enzymatic manner. Furthermore, excessive free active iron can cause a Fenton reaction with free radicals, leading to oxidative damage to the cell membrane (2). Ferroptosis is mainly determined by the balance of the antioxidant mechanism. Glutathione peroxidase 4 (GPX4), as an antioxidant enzyme, can reduce ferroptosis by converting toxic lipid peroxides into corresponding non-toxic alcohols. Notably, GPX4 activity is affected by glutathione (GSH) and the trace element selenium (3). In addition, transcription factors, such as activating transcription factor 4 (ATF4), nuclear factor erythroid 2-related factor 2 (Nrf2), and p53, can exert antioxidant effects by regulating the GSH/GPX4 pathway (4, 5).

The ovary is a female gonad that mainly produces eggs, ovulates, and secrete sex hormones, collectively known as the reproductive and endocrine functions of the ovary. Follicle is the basic functional unit of human reproduction. Follicle is mainly composed of oocytes and GC, which are interconnected through gap junctions and paracrine pathways. GC provide oocytes with ATP and small molecular energy metabolites required for growth and development through the above pathways (6). Therefore, the number and function of germ cells are closely related to the normal function of the ovary. Both genetic and environmental factors impact the fate of germ cells. Some studies have shown that oxidative stress is the main cause of many female reproductive disorders, which can decrease oocyte quality (7). The oxidative modification of lipids in the membrane bimolecular layer (especially lipid peroxidation) significantly regulates oxidative stress in cells (8). Oxidative stress and an inflammatory state associated with ferroptosis exist in the ovarian GC of patients with PCOS and endometriosis (EMs) (9, 10). Meanwhile, oxidative damage and chronic inflammation are the classic etiologies of ovarian aging (7, 11). As a result, many scholars have studied whether ferroptosis plays a role in this process and how ovarian function can be improved. In addition, the emergence of ferroptosis has made many researchers see the hope of inhibiting the growth of tumor cells due to the infinite growth characteristics of tumor cells. As a result, several mechanism studies have been conducted to improve clinical diagnosis, prediction, and treatment.

In summary, several key and common environmental factors affecting the fate of germ cells, such as metabolism, inflammation, and oxidative stress, are associated with the existing ferroptosis effect. As a result, research on the role of ferroptosis in the female reproductive system has gradually attracted much attention. Therefore, a phased summary and critical analysis of this research progress are necessary to clarify the main findings, problems, and challenges around this topic for future research. This study aimed to analyze the classical regulatory mechanism of ferroptosis and its role in the development and treatment of ovarian diseases.

## 2 The discovery of ferroptosis

The concept of ferroptosis was formally proposed by Dixon in 2012 (8). Yang and Stockwell (12) investigated the mechanism of action of small molecule compounds (Erastin and RSL3) with a selective lethal effect on RAS mutant cell lines, and discovered that the two compounds could kill cancer cells by increasing intracellular iron content and accumulating oxidizing substances. Moreover, this particular form of cell death can be inhibited by iron-chelating agents or antioxidants (which capture lipophilic free radicals). Dixon et al. (8) studied the unique morphological, biochemical, and genetic characteristics involved in erastin-induced cell death and found that: morphologically, mitochondrial shrinkage, ridge reduction, disappearance, and membrane density increase; biochemical aspects showed intracellular iron deposition and lipid peroxidation; hereditary evidence demonstrated that erastin-induced cell death is regulated by a specific genetic network (genes related to iron metabolism). Finally, the process was named “ferroptosis” due to the unique characterization of this cell death pattern and its demand for iron. Erastin can selectively destroy tumor cells or protect non-tumor cells exposed to specific oxidative conditions by manipulating ferroptosis.

## 3 Ferroptosis regulation mechanism

**Figure 1:**
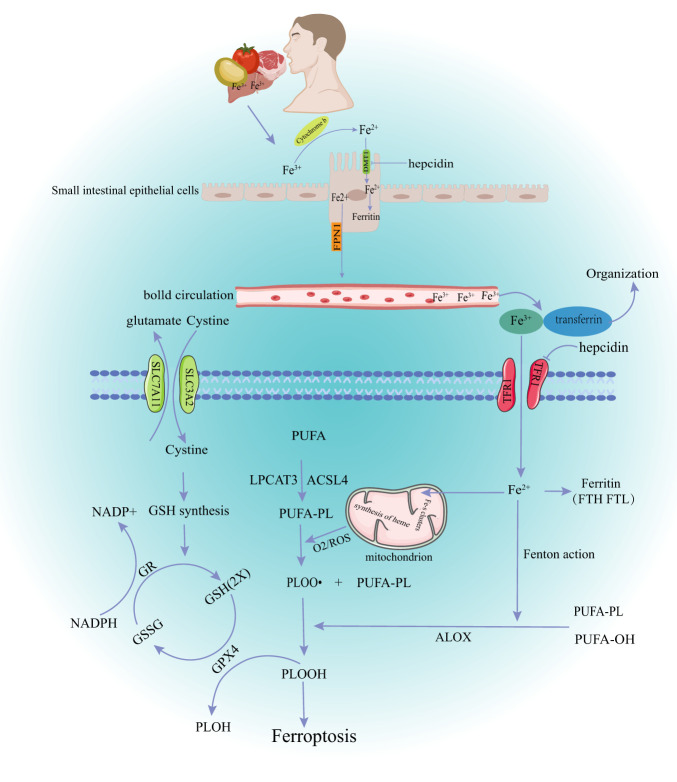
The main mechanism of ferroptosis. DMT1, divalent metal transporter 1; FPN1, ferroportin1; TFR1, Transferrin Receptor 1; GSH, glutathione; GR, Glutathion Reductase; GSSH, oxidized glutathione; GPX4, glutathione peroxidase 4; NADPH, Nicotinamide Adenine Dinucleotide Phosphate; ROS, reactive oxygen species; PUPA, polyunsaturated fatty acid; LPCAT3, Recombinant Lysophosphatidylcholine Acyltransferase 3; ACSL4,acyl-CoA synthetase long-chain family member 4; PLOOH, phospholipid hydroperoxide glutathione peroxidase.

Ferroptosis is a controlled iron-dependent lipid peroxidation cell death controlled by many metabolic pathways, such as redox reactions, iron homeostasis, glutathione metabolism and lipids (**Figure 1**).

### 3.1 Iron metabolism and ferroptosis

Dietary iron from various sources, such as meat, fish, and poultry is the main source of iron in the body under physiological conditions (13). Iron is mainly absorbed in the upper part of the small intestine. The human body has developed precise mechanisms for keeping iron levels in balance (iron homeostasis). Briefly, most Fe^3+^ in food is first reduced to Fe^2+^ by reductases, such as duodenal cytochrome B, then transported into cells through the divalent metal transporter 1 (DMT1) on the apical membrane of intestinal epithelial cells. Some iron entering the cell goes to the blood through the basolateral membrane, and the rest is temporarily stored in the cell as ferritin. Ferroportin 1 (FPN1), made by the SLC40A1 gene, is the only ferrous export protein found in mammals. Fe^2+^ leaves the intestinal epithelial cells through the pathway mediated by FPN1 when the intracellular iron is too high (14). Subsequently, Fe^2+^ is oxidized to Fe^3+^ and tightly bound to transferrin (TF) at a ratio of 2:1. TF is mainly present in the plasma, which binds to the cell surface transferrin receptor 1 (TfR1) to supply the iron needed by most tissues of the body (15). Besides the dietary iron absorbed by intestinal cells, the body also releases iron through iron storage tissues, such as the liver and spleen (16).

The body’s system of checks and balances keeps iron levels in a dynamic balance under normal conditions. Hepcidin (HEPC), which comes from the liver, can “sense” how much iron is in the body, stop intestinal absorption of iron and prevent iron release. Serum iron levels are reduced by negatively regulating the expression of duodenal iron transporters (DMT1 and FPN1) and TfR1 (17). Iron metabolism is also associated with intracellular post-transcriptional regulation. This mechanism is mediated by the central regulator of cellular iron metabolism, iron regulatory proteins (IRP1 and IRP2). IRP can bind to the iron response element (IRES) in mRNA encoding key iron regulatory proteins, including DMT1, ferritin heavy chain (FTH), TfR1, and ferritin, thereby stabilizing iron absorption, utilization, storage, and output (18). Furthermore, increased expression of hypoxia-inducible factor-2 (HIF-2), autophagy-related gene (ATG)-activated nuclear receptor coactivator 4 (NCOA4), and heme oxygenase 1 (HO-1) can regulate iron level changes in the body via the IRP pathway, ferritin autophagy, and senescent erythrocyte heme degradation (19–21). Iron in cells exists in two different forms during this regulation (Fe^2+^ and Fe^3+^). The redox reactions and other pathophysiological processes that these two valence iron ions take part in depend on the transfer of electrons between them. Fe^3+^ is relatively stable and binds to TF mainly for iron storage and transport. Fe^2+^, free iron ions, forms an unstable iron pool (LIP) in cells, which is transferred to mitochondria and participates in the formation of iron-dependent protein complexes, iron-sulfur clusters, heme, and other metabolic processes. Excessive Fe^2+^ can react with lipid radicals and H_2_O_2_ to produce a large amount of reactive oxygen species (ROS), which can damage membrane lipids, proteins, DNA, and other biological macromolecules, causing ferroptosis (22).

### 3.2 Glutathione metabolism and ferroptosis

GPX4 is an antioxidant enzyme found in mammalian cells. GPX4 prevents ferroptosis by changing toxic phospholipid hydrogen peroxide (PLOOH) into non-toxic alcohols (3). GSH is an essential cofactor of GPX4. GPX4 can turn lipid peroxides into hydroxyl compounds and oxidize glutathione. The cell membrane cystine/glutamate transporter (System Xc-, composed of two subunits of SLC7A11 and SLC3A2L) can take extracellular cystine into the cell and transport glutamate out of the cell when GSH is consumed in large quantities. Cystine is used by the cell to make GSH, which is crucial for keeping the redox balance inside and outside the cell (23). GPX4 activity depends on how well the XC-GSH-GPX4 axis works and other factors that control it. Selenium (Se) is an essential trace element for the formation of the selenoprotein glutathione peroxidase. Selenium directly affects GPX4 activity and cell sensitivity to ferroptosis (24). Selenium participates in GPX4 maturation through mevalonate pathway. However, statins can block this pathway (25). Some transcription factors can also regulate the relationship between glutathione metabolism and ferroptosis. ATF4 and Nrf2 mediate the expression of solute carrier family member 11 (SLC7A11) under stress conditions, protecting cells from ferroptosis induced by cystine starvation and excessive accumulation of ROS (4). SLC7A11 is a target of the tumor suppressor gene p53. Overexpression of some tumor genes can promote ubiquitination and degradation of p53, up-regulate SLC7A11, GPX4, antioxidant enzymes superoxide dismutase 1 (SOD-1), and SOD-2, and accelerate tumor progression by inhibiting ferroptosis in tumor cells. This also provides a target mechanism for tumor therapy (5). Recent studies have shown that the GTP cyclohydrolase-1-Tetrahydrobiopterin (GCH1-BH4) and FSP1-CoQ10-NAD (P) H pathways can inhibit ferroptosis in a way that is both parallel and synergistic with the traditional GSH/GPX4 antioxidant pathway (26, 27).

### 3.3 Lipid peroxidation and ferroptosis

Peroxidation of polyunsaturated fatty acid phospholipids (PUFA-PLs) in the plasma membrane is a major cause of ferroptosis (28). PUFA-PLs form carbon-centered phospholipid free radicals (PL•) after losing diallyl hydrogen atoms. PL•reacts with molecular oxygen to form phospholipid peroxide radicals (PLOO•), which can remove and bind hydrogen from adjacent PUFA-PLs to form lipid peroxides (PL-OOH). A large amount of PL-OOH and lipid radicals [PLOO• and alkoxyphospholipid radicals (PLO•)] can react with PUFA-PLs again if it is not converted to the corresponding alcohol by GPX4, leading to PLOOH accumulation (29). Several toxic oxides produced by this chain reaction can destroy the fluidity and structural stability of cell membranes, leading to cell rupture and death. Lipid peroxidation can also be achieved through enzymatic methods. Some lipoxygenases (LOX) (dioxygenases targeting polyunsaturated fatty acid phospholipids) can directly oxidize polyunsaturated fatty acids in biofilms, thus inducing ferroptosis (30). The non-enzymatic lipid peroxidation is mainly induced by excessive ferrous ions (Fe^2+^) or spontaneous occurrence of lipid radicals or hydroxyl radicals (•OH). Phospholipid peroxy radicals are relatively unstable and can react with Fe^2+^, resulting in a chain expansion of phospholipid peroxidation, thus triggering ferroptosis signals (2). In summary, the level of lipid peroxidation in cells depends on the polyunsaturated fatty acids in the lipids. Acyl-CoA synthetase long-chain family member 4 (ACSL4) and lysophosphatidylcholine acyltransferase 3 (LPCAT3) can increase phospholipid PUFA content. Therefore, increased expression or activity of ACSL4 and LPCAT3 may promote ferroptosis in various pathophysiological contexts. Many studies have explored the treatment of different diseases based on the above mechanisms. For example, studies have explored how ACSL4 inhibitors can improve organ ischemia-reperfusion injury and how LPCAT3 inhibitors can reshape the polyunsaturated phospholipid content of human cells to prevent ferroptosis (31, 32).

### 3.4 Transcription factors and ferroptosis

P53 and Nrf2, as widely reported transcription factors, participate in the metabolism of iron and reactive oxygen species by regulating SLC7A11, arachidonate 12-lipoxygenase (ALOX12) gene, NOQ1, HO-1, heme type I fluorosynthase and the frontotemporal hairline (FTHL) gene (33). However, recent studies have reported other transcription factors, including forkhead box O3a (FoxO3a), which participates in cell metabolism, mitochondrial dysfunction, and oxidative stress. Glucose deprivation activates AMPK/FoxO3a by binding to FoxO3a and inhibiting SLC7A11 expression, thus preventing erastin-induced ferroptosis. The absence of FoxO3a promotes ferroptosis through mitochondrial membrane potential hyperpolarization, oxygen consumption, and lipid peroxidation accumulation (34). Furthermore, activation transcription factor 3 (Atf3) reduces Acsl4m6 A modification level by up-regulating FTO expression, thus inhibiting ferroptosis (35). Repressor element 1-silencing transcription factor (REST) can regulate gene expression under hypoxia. REST knockdown can decrease ferroptosis marker GPX4 and significantly up-regulate ACSL4 in hypoxia-injured renal tubular epithelial cells (RTECs) rather than apoptosis-related proteins (Bcl-2 and Bax) (36). Furthermore, GPX4 is the downstream target of Kruppel-like factor11 (KLF11). In addition, the transcription factor KLF11 inhibits the proliferation of lung adenocarcinoma (LUAD) cells and promotes their chemosensitivity by participating in the GPX4-related ferroptosis pathway (37). The environment in which these transcription factors operate, such as energy metabolism and oxidative stress, is a common cause of most diseases, including ovarian tumors, POI, and PCOS. Besides, mitochondrial dysfunction, hypoxia, and methylation of genes are closely related to ovarian dysfunction (38–40). Therefore, further studies should confirm whether these transcription factors participate in ferroptosis regulation in ovarian diseases.

## 4 The relationship between ferroptosis and ovarian diseases

### 4.1 Ovarian aging

Ovarian aging is the process of gradual decline of ovarian function. There are two main types of ovarian aging: physiological and pathological. Physiological ovarian aging (NOA) is the gradual loss of ovarian reserve function in older women until they reach menopause while pathological ovarian aging is the premature ovarian failure caused by various pathogenic factors, including decreased ovarian reserve (DOR), ovarian insufficiency (POI) and poor ovarian response (POR) (41). Ferroptosis occurs during ovarian aging. Zheng et al. (42) showed that ACSL4 expression and ferroptosis increased in the ovaries of aged rats. The 4-hydroxy-2-nonenal (HNE) is the most sensitive marker. Besides, Zheng et al. tested five different monoclonal antibodies against HNE-modified proteins and found that HNEJ-1 is the most promising way to track ferroptosis in isolated tissues and proteins. This result indicates that ferroptosis is involved in physiological aging. However, only a few studies have explored the physiological significance of ferroptosis due to the lack of easy-to-implement and reliable methods and tools for directly monitoring ferroptosis in physiological environments. A study (43) confirmed that Tf, ferritin (Ft)) and TfR1 are abnormally up-regulated in the NOA rats based on proteomics analysis, *in vivo*, and *in vitro* research. This results in increased levels of active iron and total iron, nitrite/nitrate, 3-nitrotyrosine and HNE in aging ovaries. The ferroptosis-induced aging of the ovaries is also related to hormone secretion, which is an important function of germ cells. Ferritin inhibits the biosynthesis of estradiol in ovarian granulosa cells *in vitro* by up-regulating NF-κB and inducible nitric oxide synthase (iNOS). An adenovirus carrying ferritin light chain/heavy chain and transferrin can up-regulate NF-κB/INOS and down-regulate Nrf2/GPX4 in the ovaries of 3-month-old rats, confirming that oxidative stress-inflammatory response is mediated by iron homeostasis disorder in ovarian aging. Furthermore, Basonuclin 1 (BNC1) participates in follicular development, lipid metabolism, and redox homeostasis of oocytes (44). Therefore, BNC1 gene defects can lead to premature follicular activation and excessive atresia. Further studies have found that BNC1 deficiency triggers ferroptosis of oocytes through the Nrf2-YAP pathway, thus decreasing ovarian reserve. Also, inhibition of the YAP signal or ferroptosis can significantly rescue POI caused by BNC1 mutation. In addition, animal experiments have found that ferroptosis, cell oxidation, and vascular endothelial growth factor (VEGF) in primordial follicles are significantly increased in the ovaries of obese mice, suggesting that ferroptosis can activate immature ovarian follicles. Primordial follicle depletion is characterized by the death and activation of primordial follicles (45). Obesity has become a modern disease due to the changes in modern work and eating habits and thus may aggravate reproductive endocrine problems. Therefore, future studies should assess the relationship among ferroptosis, energy metabolism, and abnormal body fat rate.

### 4.2 Polycystic ovarian syndrome (PCOS)

PCOS is a heterogeneous gynecological disease associated with sex hormone disorders and insulin resistance. PCOS is also affected by genetic, environmental, and metabolic factors (46). PCOS is mainly characterized by ovarian granulosa cell dysfunction (47). The overexpression of MIR-93-5p in GC of PCOS patients can inhibit the expression of GPX4, SLC7A11, and Nrf2, and promote the accumulation of lipid reactive oxygen species and MDA, leading to apoptosis and ferroptosis. However, miR-93-5p silencing can prevent GC dysfunction (10). High homocysteine (Hcy) levels are associated with insulin resistance and sex hormone levels. Shi et al. found that the expression of ASCL4 and DMT1, which regulate lipid metabolism and iron metabolism, is significantly higher in Hcy-treated KGN cells than in the control group (48). These studies have shown that GC ferroptosis promotes PCOS development. In addition, PCOS patients are characterized by mitochondrial dysfunction. Mitochondrial dysfunction increases oxidative stress levels, causing or aggravating hyperandrogenism, insulin resistance, and obesity, interfering with follicular development, and thus affecting the menstrual and reproductive functions of PCOS women. As a result, some researchers (49) have shown that the activation of ferroptosis may be associated with an abnormal pregnancy (abortion) in PCOS patients. Animal tests have also shown that ferroptosis can occur in the uterus of pregnant women with PCOS, making the endometrium less receptive. Transmission electron microscopy has also shown that pregnant women with PCOS have reduced mitochondrial volume, concentrated mitochondrial membrane density, no mitochondrial ridge, and ruptured mitochondrial outer membrane (key characteristics of mitochondrial morphological changes during ferroptosis). Meanwhile, changes in mitochondrial function may also be due to ROS caused by iron elevation during ferroptosis related to mitophagy. Increased TFRC expression increases iron content, which brings NOX1 to PTEN induced putative kinase 1 (PINK1), thus promoting mitochondrial aggregation and mitophagy. As a result, cytochrome C is released into the cytoplasm to activate ACSL4 and cause lipid peroxidation, thus stopping follicular development. Therefore, the TFRC/NOX1/PINK1/ACSL4 pathway may be a potential target for PCOS (50). However, Zhang et al. recently found that n-3 polyunsaturated fatty acids (n-3 PUFAs) can activate the Hippo signaling pathway, inhibit yes-associated protein 1 (YAP1) from entering the nucleus, weaken the interaction between YAP1 and Nrf2, and increase the sensitivity of ovarian GC to ferroptosis. As a result, GC can alleviate follicular development arrest caused by abnormal proliferation of GC (51). In summary, oxidative stress, mitochondrial dysfunction, and abnormal secretion of nutritional factors associated with ferroptosis in GC affect the growth and development of ovaries. However, GC can alleviate follicular development arrest caused by excessive proliferation through ferroptosis. Therefore, further studies should confirm whether such a contradictory critical point is related to the degree of insulin resistance, body fat rate, or number of follicles in PCOS patients.

### 4.3 Ovarian cancer

Ovarian cancer (OC) is one of the most common tumors in women. OC is common in middle-aged and elderly women and seriously threatens the lives and health of women (52). Besides, the incidence rate of OC is increasing yearly. Furthermore, OC has hidden symptoms, with no clear clinical manifestations in the early stages. As a result, over 75% of OC patients are diagnosed at an advanced stage, which is related with poor therapeutic effect and high mortality (53). Therefore, the mechanism and therapeutic targets of OC occurrence and development should be determined to formulate effective treatment strategies and improve the overall survival rate of patients.

Cell death is a natural and irreversible process. Cancer cells invasively damage the body due to their infinite proliferation, loss of polarity, and decreased adhesion. However, mitochondrial dysfunction usually occurs during tumor growth since they have to make a lot of energy to maintain tumor growth (“high metabolism”). The high metabolism produces a large amount of ROS and transmits proliferation signals to promote tumor development (54). Therefore, ferroptosis is crucial in the biological process that stops rapid growth of tumor cells (55). Several tumors, including OC, grow faster when they take in and hold on to too much iron (56). High-grade serous ovarian cancer tissues and ovarian tumor-initiating cells (TICs) have reduced iron efflux pump ferritin (FPN) and increased TFR1 levels. Iron promotes cancer cell invasion through the expression of matrix metalloproteinase and the synthesis of interleukin 6 (IL-6), thereby increasing metastasis and diffusion (57). Anticancer therapies target iron inside cells through many ways. When erastin and/or the iron compound ferlixit were used to induce ferroptosis in HEY, COV318, PEO4 and A2780CP ovarian cancer cell lines, it was found that erastin treatment was accompanied by NCOA4-mediated ferritin phagocytosis and mitochondrial dysfunction in HEY cells with high intracellular unstable iron pools (58). Another study (59) found that increasing the amount of iron in ovarian cancer cells using ferric ammonium citrate (FAC) can improve ferroptosis and stop the growth of cancer cells. However, ferroptosis inducers cannot promote ferroptosis in all cancer cell lines, indicating that the genetic and metabolic susceptibility determinants limit the application of ferroptosis inducers *in vivo*.

Ferroptosis is regulated by multiple factors. However, These regulatory factors also play an important role in ovarian cancer. The p53 gene, as a typical tumor suppressor gene and a key regulator of cell metabolism, is closely related to the occurrence and development of ferroptosis (60). Overexpression of p53 can inhibit xCT and increase GSH consumption (61). The oncogene MEX3A (RNA-binding protein) destroys the stability of the p53 protein through ubiquitination, inhibits ferroptosis, and enhances tumorigenesis (62). Methylenetetrahydrofolate reductase (MTHFR) polymorphism is associated with an increased risk of gynecological cancers. Upregulation of MTHFR and heme oxygenase 1 (HMOX1) is associated with a poor prognosis in OC patients. HMOX1 is a rate-limiting enzyme that degrades heme to Fe^2+^. MTHFR can inhibit ferroptosis by blocking the ubiquitination of HMOX1. Therefore, these ferroptosis-based oncogenes can be used for the development of new therapeutic drugs. Besides, they can be used as diagnostic and predictive indicators for OC patients (63). Another study aimed to find a long non-coding RNA (LncRNA) marker associated with ferroptosis in OC and assess how it affects prognosis and clinicopathological features. In that study, lncRNAs (different between ovarian tumor tissues and normal tissues) were associated with ferroptosis genes. Finally, a prognostic risk model containing 18 LncRNAs related to ferroptosis was obtained (64). Similar studies have also found that PRNP, a ferroptosis-related gene, is significantly associated with cancer stage, main treatment outcome, and age of OC patients by mining differential genes (DEGs) of OC through public datasets. *In vitro* experiments have shown that PRNP overexpression can inhibit the proliferation, migration, and invasion of OC cells (65). Nonetheless, a prospective and multi-center study with real clinical data should assess whether the cancer-related ferroptosis gene or the ovarian cancer gene can control ferroptosis to accelerate the transformation of treatment concepts into clinical trials and improve the effectiveness of ovarian cancer treatment.

### 4.4 Other ovarian diseases

Endometriosis begins with the ectopic deposition of endometrial matrix and epithelial cells and is characterized by aggressive benign diseases. Endometrial cells form a ‘ chocolate cyst ‘ on the ovary when they are ectopic to the ovary and have undergone periodic bleeding, damage, and repair (66). Repeated bleeding of ectopic lesions can increase the concentration of free iron in chocolate cysts by 100–1000 times compared with levels in peripheral blood or other benign cysts (67). Iron overload and transferrin deficiency occur in the follicular fluid of EMs infertility patients (68). GC secretions are essential for the follicular microenvironment that controls oocyte growth and development. Some scholars have found that ferritin phagocytosis mechanisms are involved in GC ferroptosis induced by iron overload in follicular fluid *in vitro* (9). High iron content in ovarian endometriotic lesions can adversely affect adjacent GC through the free iron-mediated Fenton reaction, thereby reducing the number and quality of oocytes. This may lead to impaired fertility and adverse pregnancy outcomes (69). However, ectopic endometrial cells can acquire ferroptosis-resistant properties by up-regulating GPX4 and promoting GSH production (70). Huda I. Atiya et al. showed that a subset of ectopic endometrial cell-derived mesenchymal stem cells (enMSC) (characterized by a lack of CD10 expression) can specifically support the growth of ovarian clear cell carcinoma (OCCC). OCCC is a fatal and drug-resistant cancer that occurs in the unique microenvironment of endometriosis (71). These phenomena suggest that ectopic endometrial cells are associated with ferroptosis resistance. However, GC are highly sensitive to ferroptosis. Therefore, new therapeutic strategies should be developed based on the researched mechanism to protect GC while effectively killing ectopic endometrial cells, thereby alleviating the suffering of patients.

## 5 Regulatory mechanisms of ferroptosis in ovarian diseases

**Figure 2:**
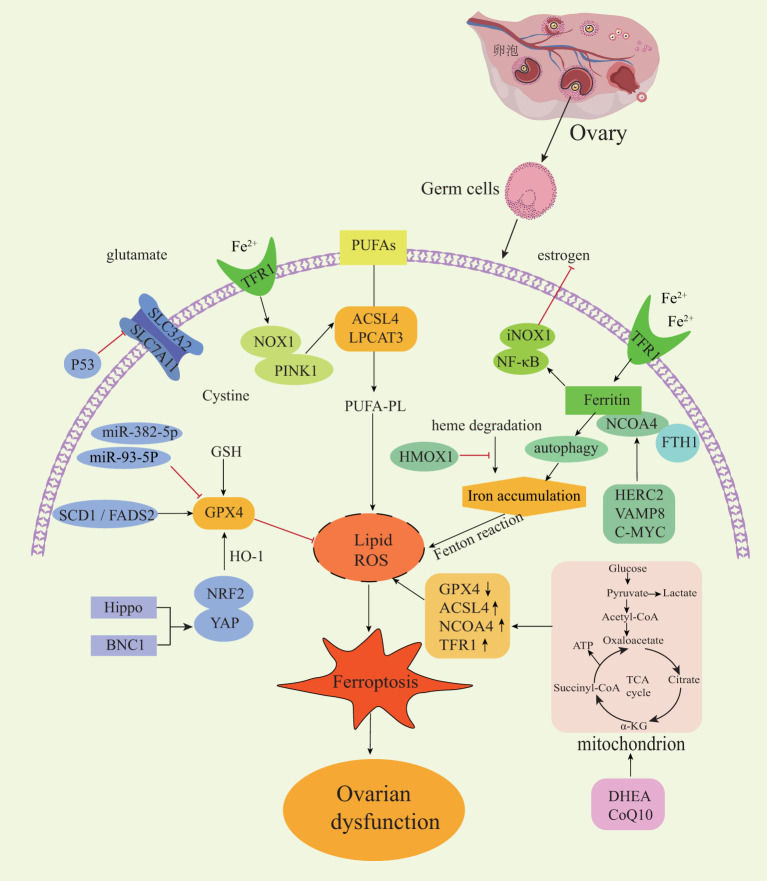
Regulatory mechanisms of ferroptosis in ovarian diseases. NOX1, NADPH oxidase 1; PINK1, PTEN induced kinase 1; NF-κB, nuclear factor kappa B; NCOA4, Nuclear receptor coactivator4; FTH1, ferritin heavy chain-1; HMOX1, heme oxygenase 1; HERC2, E3 ubiquitin ligase for NCOA4 and FBXL5; VAMP8, mediates autophagosome-lysosome fusion; SCD1, stearoyl-CoA desaturase-1; FADS2, acyl-CoA 6-desaturase; Nrf2, nuclear factor erythroid 2-related factor 2; YAP, yes-associated protein; BNC1, Basonuclin 1.

The types of ovarian diseases described in this article only differ in their disease background and predisposing factors and ultimately affect the reproductive and endocrine functions of the ovary by acting on various types of germ cells. Mitochondrial dysfunction, oxidative stress and inflammatory response are the common upstream pathogenesis of various ovarian diseases. Ferroptosis is a type of regulatory cell death that is closely related to the mitochondria, oxidative stress and inflammatory response. At present, there are several crosstalk mechanisms between classical regulatory genes of ferroptosis in ovarian diseases (**Figure 2**). Nrf2 is a key regulator of antioxidant response and a ferroptosis signaling pathway with Nrf2 as the core plays an important role in protecting cells from ferroptosis. Excessive androgen is one of the key pathogenic markers of PCOS. The impaired cell anti-ferroptosis activity and increased fetal loss rate observed in pregnant women with PCOS can be attributed to the interaction between over-activated androgen receptors and Nrf2. This interaction leads to lower levels of SLC7A11 and GPX4 proteins and higher levels of 4-HNE modified proteins in pregnant rats. These molecular changes contribute to the dysregulation of cell anti-ferroptosis mechanisms, ultimately impacting fetal development and survival in PCOS pregnancies (72). Estrogen has an antioxidant function, and estrogen deficiency can inhibit the Nrf2/GPX4 pathway to induce ferroptosis (73). Estrogen has also been found to reverse chemotherapy resistance of ovarian cancer by targeting the Nrf2/GPX4 signaling axis to induce ferroptosis in drug-resistant human epithelial ovarian cancer cells (74). In conclusion, activation of the Nrf2 signaling pathway plays a crucial role in protecting ovarian granulosa cells from oxidative damage and ferroptosis (72). This also provides a mechanism for exploring the application of other antioxidant drugs in ovarian diseases.

The Hippo signaling pathway is a highly conserved pathway across diverse species and plays a crucial role in regulating the proliferation and differentiation of various cell types (75). The Hippo pathway is particularly important in follicular development because the activation of this pathway causes changes in the nuclear localization of YAP1 to perform different regulatory functions. The crosstalk between YAP1 and Nrf2 can change the sensitivity of cells to ferroptosis. BNC1 is involved in lipid metabolism and maintaining redox homeostasis in oocytes. The dysfunction of BNC1 activates ferroptosis through the Nrf2-Hippo pathway in oocytes, resulting in POI (44). NCOA4 is a selective receptor that binds to ferritin heavy chain-1 (FTH1) to form a ferritin complex, which targets lysosomes for ‘ferritin phagocytosis’ and promotes ferritin degradation. It has been reported that the low expression of NCOA4 in cells is accompanied by an increased Fe^2+^ and oxidative stress level, a decreased GSH level. Thus, NCOA4 is recognized as a facilitator of ferroptosis, while HERC2 (an E3 ubiquitin ligase for NCOA4 and FBXL5), VAMP8 (involved in autophagosome-lysosome fusion), and C-MYC have the potential to influence ferritin autophagy by modulating NCOA4 expression. Consequently, these factors participate in regulate ferroptosis and immune escape of ovarian cancer cells, highlighting new therapeutic targets that could alleviate chemotherapy resistance among ovarian cancer patients (76–78).

Germ cells require extremely high mitochondrial dynamics to maintain physiological characteristics, and a high-load operation of the mitochondrial respiratory chain is closely related to ROS production. Metabolic reprogramming mainly occurs in the mitochondria and cytoplasm, which refers to the metabolic changes made by cells in response to various stimulus pressures. To reveal the intricate interaction between cell energy metabolism and ferroptosis in senescent germ cells, Lin et al. included 75 patients with ovarian aging. Using multi-omics analysis combined with human ovarian pathology and clinical biopsy, they found that glucose metabolism and tricarboxylic acid cycle (TCA) cycles changed in the granulosa cells of aging patients. It has been verified that supplementary metabolic reprogramming nutrients [such as dehydroepiandrosterone (DHEA) and coenzyme Q10 (CoQ10)] can cause changes in glycolysis and enhance mitochondrial oxidative phosphorylation by regulating the expression of ferroptosis-related genes. Consequently, targeting and inhibiting the ferroptotic effect on germ cells holds promise for enhancing the success rate of *in vitro* fertilization (IVF) in elderly patients (79). In addition, the relationship between ferroptosis and inflammation is a research hotspot. NF-κB plays a key role in regulating the immune response to infection, and thus it has a chronic activity in some inflammatory diseases. A previous study found that abnormally up-regulated transferrin and ferritin in the ovaries of naturally aging rats can cause iron-accumulating oxidative stress and inflammatory aging through the NF-κB signaling pathway (43). Similarly, it was also found that activated NF-κB can reduce the expression of GPX4, SLC7A11 and Nrf2 and promote GC cell apoptosis and ferroptosis in PCOS disease, and thus it negatively impacts ovarian function (10). Inflammatory injury, repair and fibrosis are closely related. Although it has been reported that conventional chemotherapy drugs (such as cyclophosphamide (CTX) and paclitaxel) promoted the production of excessive superoxide in GC to trigger lipid peroxy-iron death, inducing decreased ovarian reserve and ovarian fibrosis in mice (80). To date, the presence of a ferroptosis-associated inflammatory regulatory mechanism in the advancement of ovarian aging and ovarian fibrosis associated with EMs has not been investigated (81).

## 6 Treatment based on ferroptosis

Ferroptosis is gradually being recognized as an adaptive process. The body can remove cells damaged by nutrient deficiency, infection or stress through ferroptosis. Therefore, ferroptosis has an inhibitory effect on tumor cells (82). Excessive ferroptosis is also closely related to autoimmune diseases, ovarian aging, polycystic ovary syndrome and other diseases (10, 42, 83). Existing studies have shown that turning on or turning off ferroptosis may help treat diseases (**Table 1**).

**Table 1:** A brief description of the molecular target about ferroptosis in its related ovary diseases.

Disease	Model	Molecular target or key pathway	Trigger mechanism of Ferroptosis	medicine	References
ovarian aging	small white follicles of laying chickens	Nrf2/HO-1	oxidative damage	rutin	(84)
ovarian aging	Ovary of rats	ACSL4	Lipid Peroxidation	~	(42)
ovarian aging	Rat Ovaries and rat granulosa cells	Transferrin/Ferritin/NF-κb	Iron Accumulation and Oxidative Inflammaging	~	(43)
Primary ovarian insufficiency	Mice Ovary and Oocytes	BNC1/Nrf2-YAP	lipid metabolism and redox homeostasis	ferrostatin−1	(44)
PCOS	Mice Ovary Human ovarian granulosa cell tumor cells (KGN cells)	miR-93-5p/NF-kB	apoptosis and ferroptosis	~	(10)
PCOS	KGN cell	TET levels and DNA methylation/GPX4, SLC7A11, ASCL4 and DMT1.	oxidative stress	ferrostatin−1	(48)
PCOS	Mice Ovary KGN cells	TFRC/NOX1/PINK1/ACSL4	Mitophagy and lipid peroxidation	~	(50)
PCOS	Rat Ovaries and Rat Ovarian Granulosa Cells	NFR1, GPX4, NF-κB and MAPK/ERK	oxidative stress, inflammation, and apoptosis	Cryptotanshinone (CRY)	(85)
PCOS	Mice Ovary	GPX4/SIRT3/AMPK/mTOR	mitochondrial oxidative pathway	Metformin	(86)
ovarian cancer	ascites-derived ovarian cancer cells; the clinical samples (tumor, omentum, and ascites from OvCa patients)	SCD1/FADS2/GPX4/GSH/GSSG	iron-mediated lipid peroxidation and mitochondrial dysfunction	~	(87)
ovarian cancer	Ovarian cancer cell line SKOV-3; Tumor tissues of ovarian cancer patients	MiR-382-5p/SLC7A11	accumulation of Fe^2+^; iron and lipid reactive oxygen species (ROS)	Lidocaine	(19)
ovarian cancer	Tumor tissues of ovarian cancer patients; Human ovarian cancer cell lines HO8910 and SKOV3	miR-424-5p/ACSL4	Lipid peroxidation	erastin and RSL3	(88)
ovarian cancer	Human ovarian cancer cell lines TOV-21G/ES-2/RMG-2/RMG-V/OVCA432/OVCA429/OVCA420	TAZ/ANGPTL4/NOX2	Lipid peroxidation;sensitivity to the erastin-induced ferroptosis	~	(89)
ovarian cancer	high-grade serous ovarian tumors (HGSOC) and associated malignant ascites; Platinum-Tolerant mice and SKOV3/OVCAR3 Cells	Frizzled-7/GPX4	redox homeostasis	~	(90)
ovarian cancer	Ovarian Cancer Xenograft Mouse; human ovarian cancer cells lines (SKOV3/ES2/OVCAR3/OVCAR8)	glutathione peroxidase (GPx); cystine/glutamate antiporter system Xc (xCT)	ROS accumulation	Selenium-Chrysin Polyurea Dendrimer Nanoformulation/sodium selenite	(91)

### 6.1 Role of ferroptosis in cancer therapy

At present, the first-line chemotherapy for ovarian cancer is platinum drugs combined with paclitaxel and targeted drugs bevacizumab and PARP inhibitors are often used for maintenance therapy. Because many people who receive chemotherapy have primary or secondary resistance to it, the treatment does not work as effectively as it should (92). Studies have shown that combining ferroptosis agonists with cisplatin (CDDP) or PARP inhibitors can inhibit ovarian cancer cell growth and metastasis. It is reasonable to ascertain the direction of the treatment of these drug-resistant tumors using ferroptosis (93, 94). With the continuous expansion of the treatment direction for ovarian cancer, new ferroptosis-based drugs, molecules and targets are constantly being developed.

#### 6.1.1 Molecular targets

Peritoneal metastasis is the most common type of cancer cell growth in ovarian cancer patients, and it is strongly linked to a poor prognosis (95). Notably, the amount of fatty acid desaturation is critical in keeping most malignant tumors dry and aggressive (96). Lipidomics analysis showed that elevated unsaturated fatty acids (UFAs) were positively correlated with SCD1/FADS2 (two key fatty acid desaturases) levels and the carcinogenic ability of ovarian cancer cells (OvCa) (87). Genetic deletion of SCD1/FADS2 or direct blockage with a drug downregulates GPX4 and GSH/GSSG ratios. This destroys the cell/mitochondrial redox balance and accelerates cell death through iron-mediated lipid peroxidation, providing a promising chemotherapy strategy for peritoneal metastases of epithelial ovarian cancer. MiRNA interacts with target gene mRNA and regulates the translation of functional proteins and various biological processes during carcinogenesis. Some researchers have found that miR-382-5p expression is down-regulated and SLC7A11 expression is up-regulated in ovarian cancer tissues, and lidocaine targets the miR-382-5p/SLC7A11 axis (which up-regulates miR-382-5p expression and down-regulates SLC7A11 expression) to promote ferroptosis in ovarian cancer cells and exert anti-tumor activity (97). Lidocaine can also increase the expression of ACSL4 by directly knocking down miR-424-5p to promote lipid peroxidation in ovarian cancer cells and increase their sensitivity to the ferroptosis activators erastin and RSL3 (88). Some marker genes can also be used to help choose tumors that are most likely to respond to ferroptosis induction therapy. Ovarian cancer with high expression of PDZ-binding motif (TAZ) and Angiopoietin-Like 4 (ANGPTL4), which is sensitive to ferroptosis, is a good example of a tumor that is likely to respond to this therapy. Chemotherapy resistance to such ovarian cancer can be eliminated using ferroptosis activators (89). In addition, Wang et al. (90) found that the expression of the Wnt receptor Frizzled-7 (FZD7) was increased in carboplatin-resistant cells and tumor tissues. Overexpression of FZD7 activates the carcinogenic factor Tp63, drives the up-regulation of GPX4, and protects cells from chemotherapy-induced oxidative stress. This finding reveals that Frizzled-7-labeled platinum-resistant cancer cells have GSH metabolic changes and also provides a breakthrough for the treatment of ovarian cancer by inducing ferroptosis.

#### 6.1.2 Drugs

Progressive drug development is essential for the treatment of clinical patients. Systematic studies of highly effective anticancer drugs provide valuable insights into the discovery of new anticancer drugs and have great potential for the expansion of the anticancer direction of existing drugs. Sorafenib — as a ferroptosis-related clinical drug — has been approved by the United States Food and Drug Administration for the treatment of ovarian cancer (98). A multicenter, double-blind, randomized, placebo-controlled phase 2 trial assessed the efficacy of sorafenib plus topotecan as a maintenance therapy for ovarian cancer that was resistant or unresponsive to platinum. Statistically and clinically, the results improved the progression-free survival of women with ovarian cancer who were resistant to platinum (99). Chen et al. (100) developed a hybrid compound called linoleic acid (LA)-glucosamine (GlcN) (LA-GlcN) as an effective treatment for high-grade serous ovarian cancer (HGSOC). The unique feature of this compound is that GlcN specifically targets and recognizes the overexpressed glucose transporter 1 (GLUT 1) in tumor cells, thereby enhancing the uptake of LA-GlcN. Since HGSOC cells contain approximately five times more iron than normal ovarian cells, the presence of unsaturated LA triggers ferroptosis, a process involving iron-dependent lipid peroxidation that leads to the breakdown of fats at a rapid pace. Norcantharidin (NCTD) is a demethylated form of cantharidin, which is widely used in clinical practice as an optional anticancer drug. *In vitro* studies showed that norcantharidin significantly raised the levels of MDA and Fe^2+^ and caused ferroptosis in ovarian cancer cells by stopping Nrf2/HO-1 signaling. Its use in ovarian cancer was initially expanded (101). Since cancer treatment using traditional Chinese medicine has gradually attracted attention, some experts have isolated the bioactive protein MAP30 from bitter melon seeds and administered it simultaneously with cisplatin. MAP30 has been shown to activate AMP-activated protein kinase (AMPK) signaling via CaMKK-, resulting in a synergistic effect on cisplatin-induced ovarian cancer cytotoxicity. Notably, blood tests showed that MAP30 did not impair liver or kidney function in MAP30-treated mice (102). Experts recently highlighted that sodium molybdate (Na2MoO4) is a molybdenum compound that is soluble and easily absorbed by organisms. Na2MoO4 can not only induce the increase in the unstable iron pool in ovarian cancer cells but also induce the depletion of GSH by mediating the production of nitric oxide (NO). Meanwhile, NO causes apoptosis in ovarian cancer cells by inhibiting mitochondrial aconitase activity and ATP production. Due to its multiple inhibitions of cell proliferation and activity, it is a top candidate drug for ovarian cancer (103).

With the increase in drug categories, some studies have focused on improving the safety, absorption efficiency and action time of drugs to strive for perfection. The enhanced permeability and retention (EPR) effect of nanotechnology shows the advantages of drug solubility, effective systemic circulation and tumor targeting. In their study, Gao et al. (104) developed nanoparticle micelles composed of arachidonic acid-conjugated amphiphilic copolymers to encapsulate RSL3. These micelles, when exposed to free radicals present in the tumor microenvironment, facilitate the swift release of RSL3, which targets the protein GPX4. *In vitro* and *in vivo* experiments demonstrated that drug-loaded micelles significantly enhanced the efficacy of RSL3 in inducing ferroptosis in drug-resistant cancer cells, thereby weakening the multidrug resistance (MDR) of anticancer drugs. Other studies investigated the two mechanisms of action of high-dose sodium selenite and selenium-containing chrysin (SeChry) in ovarian cancer (91, 105). As a competitive inhibitor of xCT, it promotes GSH consumption while inhibiting the expression of the antioxidant H2S-producing enzyme cystathionine-synthase (CBS) to achieve oxidative stress toxicity. As a vehicle to deliver SeChry to its target, it was inserted in folic acid-targeted polyurea dendrimer fourth-generation (PUREG4-FA) nanoparticles. The delivery of Sechry to ovarian cancer cells was more targeted when nanoparticles were used. The drug concentration in the tumor reached the therapeutic threshold without harming adjacent normal cells.

### 6.2 Inhibition of ferroptosis exerts a therapeutic effect

Inhibition of ferroptosis is undoubtedly an effective treatment option for functional disorders involving ferroptosis. Ferrostatin-1 (Fer-1) is the first aromatic amine found to effectively inhibit ferroptosis and lipid peroxidation accumulation and protect cells from multiple stresses and/or toxic chemicals. Recently, it was demonstrated that ferroptosis blockade significantly improved the estrous cycle disorder and prolonged the estrous cycle of primary ovarian aging by intraperitoneal injection of Fer-1 in Bnc1 mutant mice, and the ratio of oxidized to reduced lipids in the ovary was significantly reduced. One of the most important ways that ovaries age is through oxidative free radicals, and rutin has anti-inflammatory and antioxidant effects (44). Wu et al. (84) found that rutin activated the Nrf2/HO-1 signaling pathway to reduce oxidative stress caused by ferroptosis in the ovaries of naturally aging chickens. This suggests that rutin plays a protective role in ovarian function in elderly laying hens and prolongs the laying period. As a traditional Chinese medicine, electroacupuncture (EA) can inhibit oxidative stress and ferroptosis in the ovaries of premature ovarian failure mice by improving fibrosis and atresia follicles and increasing hormone levels in POF mice, thereby promoting follicular maturation, EA also offers the benefit of minimal side effects (106). Despite the contrasting follicle counts observed in PCOS and ovarian aging, both conditions are affected by oxidative stress and ferroptosis, leading to a significant decline in the quality of oocytes and granulosa cells. In the KGN cell model of PCOS, Fer-1 was found to inhibit the apoptosis, oxidative stress and ferroptosis of KGN cells. This protective effect may be achieved by enhancing the Tet enzyme (dioxygenase) and DNA methylation. This provides an experimental basis for the application of Fer-1 as a potential therapeutic drug in the clinical treatment of PCOS (48). Cryptotanshinone is an extract of Danshen (*Salvia miltiorrhiza* Bunge). *Salvia miltiorrhiza* is a pleiotropic plant recognized in traditional Chinese medicine for the treatment of various diseases (107). Cryptotanshinone can inhibit oxidative stress, inflammation and ferroptosis by activating MAPK/ERK signaling to prevent ovarian tissue damage in PCOS (108). Adverse pregnancy outcomes caused by endocrine disorders in PCOS are also common clinical problems. Because metformin can improve insulin resistance and obesity, it is commonly used in the treatment of PCOS. It was recently reported that metformin improves PCOS in mice by inhibiting ovarian ferroptosis, which has expanded the new mechanism of this drug. This is the so-called new mechanism of old drugs (85). Other animal experiments have found that an antioxidant N-acetylcysteine can activate the SLC7A11-GSH-GPX4 axis and regulate iron metabolism, suggesting that antioxidants based on the mechanism of ferroptosis may be a good measure to prevent PCOS complicated with pregnancy loss (86).

## 7 Summary

Ferroptosis is a process involving abnormal metabolism of iron, glutathione and lipids and is regulated by various mechanisms. It is characterized by redox imbalance caused by abnormal metabolic processes, which ultimately leads to cell injury and death. The infinitely rapid proliferation of ovarian tumor cells and the high metabolic characteristics of germ cells require large amounts of energy from the mitochondria, which are the main sources of ROS. Once the imbalance between oxidation and antioxidation is established, it is easy to stimulate waterfall oxidative stress. The free radical theory that ROS cause DNA damage in germ cells is also one of the main pathogeneses of ovarian aging and dysfunction. The simultaneous development of multiple follicles in PCOS patients requires energy, and endocrine disorders (such as insulin resistance, elevated androgen hormones, etc.) are accompanied by abnormal glucose and lipid metabolism. The periodic damage and repair of the ovary in endometriosis are in a continuous inflammatory state. Therefore, numerous studies have been reasonably carried out on ferroptosis in female ovarian diseases. Ovarian tumor studies found that some molecules, such as SCD1/FADS2, miR-382-5p, Frizzled-7, etc., will be used as targets for future intervention in clinical treatment. It can also improve the chemoresistance of tumor cells by increasing the sensitivity of ovarian tumor cells to ferroptosis and guide clinical decision-making by analyzing the tumor risk prognostic model of ferroptosis-related genes in ovarian tumor tissues. Studies on benign ovarian diseases have found that the ferroptotic effect of ovarian granulosa cells affects the growth and development of oocytes, ultimately influencing ovarian function. The mechanism mainly involves Nrf2/GPX4, Hippo, NF-κB, NCOA4, ferritin autophagy, energy metabolism reprogramming, etc. Interestingly, some drugs based on the study of the regulatory mechanisms of ferroptosis in ovarian diseases, such as cryptotanshinone, N-acetylcysteine, rutin etc., has significantly improved ovarian function.

Although ferroptosis and its related regulatory drugs have great potential in ovarian diseases, current research on ferroptosis in ovarian diseases is still in its infancy. For example, some ferroptosis-related predictors, targets and models are still at the level of multi-omics sequencing and basic experimental verification. Besides, there is a huge need for prospective experimental big data to augment the application of ferroptosis and its related regulatory drugs in clinical practice. There are many types of ovarian cancer. Moreover, research should not only focus on the common types of high clinical incidence but should also be more comprehensive to explore the relationship between different subtypes of ovarian cancer and ferroptosis, to carry out mechanism-regulated therapeutic intervention more accurately. Since mitochondria are the main source of cell energy supply, the relationship between ferroptosis and metabolic reprogramming may also play a role in other highly metabolic ovarian diseases such as PCOS and ovarian tumors, in addition to ovarian aging. Furthermore, ferroptosis of granulosa cells in PCOS has different effects on ovarian function, which may be related to the severity of PCOS and individual heterogeneity. Notably, close attention should be paid to the methods that can improve ferroptosis resistance of ectopic endometrial cells in the ovary while reducing the iron damage to adjacent ovarian granulosa cells and oocytes. In recent studies, several transcription factors, including FoxO3a, Atf3, REST, and KLF11, have been suggested to play a regulatory role in ferroptosis. These studies have specifically investigated the involvement of these transcription factors in conditions such as cerebral ischemia-reperfusion injury, acute kidney injury, and lung adenocarcinoma (34–37). Hence, further research on these transcription factors in ovarian diseases may provide some new ideas for solving clinical problems. Despite considerable advancements in the investigation of ferroptosis in animal and cell models of ovarian diseases, there remain some unresolved issues that must be addressed before the findings from experimental studies can be effectively translated into clinical treatments for ovarian diseases.

## Author contributions

YY and YL conceived the study and designed the contents. YY and BW drew patterns for the paper. YY and BW wrote the manuscript. YY and YL supervised the entire project. YJ and HG made a lot of contributions in the revision of the manuscript. All authors have read and agreed to the published version of the manuscript.
